# Methyl 1-(2,6-difluoro­benz­yl)-1*H*-1,2,3-triazole-4-carboxyl­ate

**DOI:** 10.1107/S1600536811006684

**Published:** 2011-03-02

**Authors:** Su-Lan Dong, Xiao-Chun Cheng

**Affiliations:** a College of Life Science and Chemical Engineering, Huaiyin Institute of Technology, Huaiyin 223003, Jiangsu, People’s Republic of China

## Abstract

In the title compound, C_11_H_9_F_2_N_3_O_2_, the triazole ring is planar, with an r.m.s. deviation of 0.0048 Å, and makes a dihedral angle of 77.3 (1)° with the benzene ring. In the crystal, weak inter­molecular C—H⋯O and C—H⋯N hydrogen bonds link the mol­ecules into chains along the *b* axis.

## Related literature

For the synthetic procedure and applications of the title compound, see: Arroyo (2007 ▶). The title compound is an inter­mediate in the preparation of the anti­convulsant drug rufinamide [systematic name 1-(2,6-difluoro­benz­yl)-1*H*-1,2,3-triazole-4-carboxamide], see: Meier (1986 ▶). For bond-length data, see: Allen *et al.* (1987 ▶). 
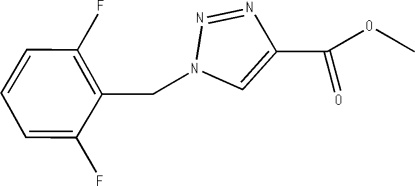


## Experimental

### 

#### Crystal data

C_11_H_9_F_2_N_3_O_2_
*M* *_r_* = 253.21
Monoclinic, 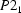

*a* = 8.4570 (17) Å
*b* = 5.4140 (11) Å
*c* = 12.125 (2) Å
β = 92.28 (3)°
*V* = 554.72 (18) Å^3^
*Z* = 2
Mo *K*α radiation
μ = 0.13 mm^−1^
*T* = 298 K
0.30 × 0.20 × 0.10 mm

#### Data collection

Enraf–Nonius CAD-4 diffractometer
Absorption correction: ψ scan (North *et al.*, 1968 ▶) *T*_min_ = 0.962, *T*_max_ = 0.987
2187 measured reflections
1146 independent reflections
1035 reflections with *I* > 2σ(*I*)
*R*_int_ = 0.031
3 standard reflections every 200 reflections  intensity decay: 1%

#### Refinement

*R*[*F* ^2^ > 2σ(*F* ^2^)] = 0.032
*wR*(*F* ^2^) = 0.088
*S* = 1.04
1146 reflections
164 parameters
1 restraint
H-atom parameters constrained
Δρ_max_ = 0.13 e Å^−3^
Δρ_min_ = −0.17 e Å^−3^

### 

Data collection: *CAD-4 Software* (Enraf–Nonius, 1989 ▶); cell refinement: *CAD-4 Software*; data reduction: *XCAD4* (Harms & Wocadlo, 1995 ▶); program(s) used to solve structure: *SHELXS97* (Sheldrick, 2008 ▶); program(s) used to refine structure: *SHELXL97* (Sheldrick, 2008 ▶); molecular graphics: *SHELXTL* (Sheldrick, 2008 ▶); software used to prepare material for publication: *SHELXTL*.

## Supplementary Material

Crystal structure: contains datablocks I, global. DOI: 10.1107/S1600536811006684/bq2275sup1.cif

Structure factors: contains datablocks I. DOI: 10.1107/S1600536811006684/bq2275Isup2.hkl

Additional supplementary materials:  crystallographic information; 3D view; checkCIF report

## Figures and Tables

**Table 1:** Hydrogen-bond geometry (Å, °)

*D*—H⋯*A*	*D*—H	H⋯*A*	*D*⋯*A*	*D*—H⋯*A*
C7—H7*B*⋯N3^i^	0.97	2.62	3.538 (4)	157
C8—H8*A*⋯O1^ii^	0.93	2.35	3.243 (3)	162

Symmetry codes: (i) 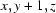; (ii) 
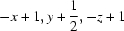
.

**Fig. 1.**
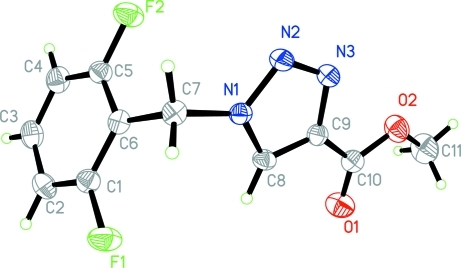
View of the title compound with the atom numbering scheme. Displacement ellipsoids are drawn at the 30% probability level.

**Fig. 2.**
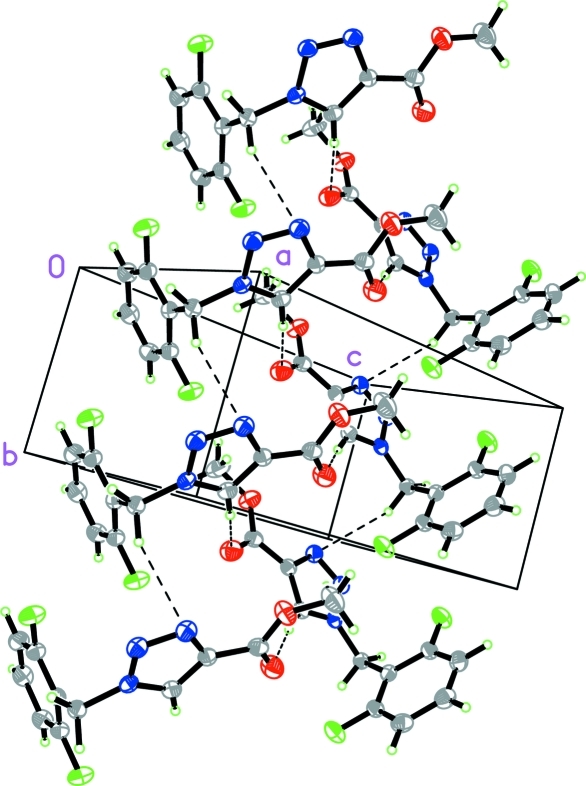
The crystal structure of (I). Dashed lines indicate C—H···N and the C—H···O hydrogen bonds.

## supplementary crystallographic information

### Comment

The title compound C_11_H_9_F_2_N_3_O_2_, (I), was synthesized by the reaction of 2,6-fluorobenzyl azide and methyl propiolate (Arroyo, 2007), and it is an important organic intermediate which is useful in preparing medicine rufinamide (Meier, 1986).

The molecular structure of (I) is shown in Fig. 1, the bond lengths and angles are within normal ranges (Allen *et al.*, 1987). For synthetic procedure, see: Meier, 1986. For background to the applications, see: Arroyo, 2007.

Ring A (C1—C6) and B (C8/C9/N1/N2/N3) are planar with r.m.s. deviations of 0.0048 ° and 0.0022 °, respectively, and the dihedral angle between them is 77.3 (1) ° (Fig.1).

As can be seen from the packing diagram (Fig.2), the crystal packing is stabilized by intermolecular C—H···O and C—H···N hydrogen bonds along the *b* axis.

### Experimental

A mixture of 2,6-fluorobenzyl azide (390 g, 1.66 mol), methyl propiolate (165 g, 1.97 mol) and methanol (2 *L*) was stirred and refluxed for 10 h. Removing of the solvent under reduced pressure gave a yellowish soil. The soil could be recrystallized using a mixture of petroleum ether and methanol (4:1) and product to be a white and spiculate soil (yield; 299 g, 51.8%, m.p. 413 K). Crystals of (I) suitable for *x*-ray diffraction were obtained by slow evaporation from methylalcohol (AR) (10 ml).

### Refinement

H atoms were positioned geometrically and constrained with C—H = 0.96, 0.97 and 0.93 Å for methyl H, methylene H and all the other H atoms, respectively, and constrained to ride on their parent atoms, with *U*_iso_(H) = *x U*_eq_(C), where *x* = 1.5 for methyl H, and *x* = 1.2 for all other H atoms.

### Crystal data

**Table:**

C_11_H_9_F_2_N_3_O_2_	*F*(000) = 260
*M**_r_* = 253.21	*D*_x_ = 1.516 Mg m^−^^3^
Monoclinic, *P*2_1_	Mo *K*α radiation, λ = 0.71073 Å
Hall symbol: P 2yb	Cell parameters from 25 reflections
*a* = 8.4570 (17) Å	θ = 10–14°
*b* = 5.4140 (11) Å	µ = 0.13 mm^−^^1^
*c* = 12.125 (2) Å	*T* = 298 K
β = 92.28 (3)°	Spiculate, colorless
*V* = 554.72 (18) Å^3^	0.30 × 0.20 × 0.10 mm
*Z* = 2	

### Data collection

**Table:**

Enraf–Nonius CAD-4 diffractometer	1035 reflections with *I* > 2σ(*I*)
Radiation source: fine-focus sealed tube	*R*_int_ = 0.031
graphite	θ_max_ = 25.4°, θ_min_ = 1.7°
ω/2θ scans	*h* = 0→10
Absorption correction: ψ scan (North *et al.*, 1968)	*k* = −6→6
*T*_min_ = 0.962, *T*_max_ = 0.987	*l* = −14→14
2187 measured reflections	3 standard reflections every 200 reflections
1146 independent reflections	intensity decay: 1%

### Refinement

**Table:**

Refinement on *F*^2^	Primary atom site location: structure-invariant direct methods
Least-squares matrix: full	Secondary atom site location: difference Fourier map
*R*[*F*^2^ > 2σ(*F*^2^)] = 0.032	Hydrogen site location: inferred from neighbouring sites
*wR*(*F*^2^) = 0.088	H-atom parameters constrained
*S* = 1.04	*w* = 1/[σ^2^(*F*_o_^2^) + (0.0542*P*)^2^ + 0.0317*P*] where *P* = (*F*_o_^2^ + 2*F*_c_^2^)/3
1146 reflections	(Δ/σ)_max_ < 0.001
164 parameters	Δρ_max_ = 0.13 e Å^−^^3^
1 restraint	Δρ_min_ = −0.16 e Å^−^^3^

### Special details

**Table:**

Geometry. All e.s.d.'s (except the e.s.d. in the dihedral angle between two l.s. planes) are estimated using the full covariance matrix. The cell e.s.d.'s are taken into account individually in the estimation of e.s.d.'s in distances, angles and torsion angles; correlations between e.s.d.'s in cell parameters are only used when they are defined by crystal symmetry. An approximate (isotropic) treatment of cell e.s.d.'s is used for estimating e.s.d.'s involving l.s. planes.
Refinement. Refinement of *F*^2^ against ALL reflections. The weighted *R*-factor *wR* and goodness of fit *S* are based on *F*^2^, conventional *R*-factors *R* are based on *F*, with *F* set to zero for negative *F*^2^. The threshold expression of *F*^2^ > σ(*F*^2^) is used only for calculating *R*-factors(gt) *etc*. and is not relevant to the choice of reflections for refinement. *R*-factors based on *F*^2^ are statistically about twice as large as those based on *F*, and *R*- factors based on ALL data will be even larger.

### Fractional atomic coordinates and isotropic or equivalent isotropic displacement parameters (Å^2^)

**Table:**

	*x*	*y*	*z*	*U*_iso_*/*U*_eq_	
N1	0.4481 (2)	0.4288 (4)	0.80667 (14)	0.0340 (5)	
F1	0.67849 (18)	0.9724 (4)	0.74993 (14)	0.0589 (5)	
O1	0.3504 (3)	0.2067 (5)	0.47974 (15)	0.0642 (6)	
C1	0.7743 (3)	0.7947 (5)	0.7942 (2)	0.0409 (6)	
F2	0.74809 (18)	0.2564 (4)	0.96110 (13)	0.0595 (5)	
N2	0.3697 (2)	0.2386 (5)	0.85061 (16)	0.0424 (5)	
O2	0.2160 (2)	−0.0844 (5)	0.56720 (16)	0.0613 (6)	
C2	0.9334 (3)	0.8063 (6)	0.7754 (2)	0.0500 (7)	
H2B	0.9745	0.9332	0.7336	0.060*	
N3	0.3078 (2)	0.1088 (5)	0.76968 (17)	0.0436 (5)	
C3	1.0299 (3)	0.6249 (7)	0.8204 (2)	0.0527 (7)	
H3B	1.1377	0.6284	0.8080	0.063*	
C4	0.9699 (3)	0.4388 (7)	0.8831 (2)	0.0517 (7)	
H4A	1.0354	0.3168	0.9137	0.062*	
C5	0.8097 (3)	0.4382 (5)	0.89946 (19)	0.0409 (6)	
C6	0.7066 (3)	0.6141 (5)	0.85681 (19)	0.0361 (5)	
C7	0.5322 (3)	0.6069 (5)	0.87801 (19)	0.0373 (6)	
H7A	0.5179	0.5633	0.9546	0.045*	
H7B	0.4873	0.7699	0.8655	0.045*	
C8	0.4362 (3)	0.4207 (6)	0.69632 (18)	0.0374 (5)	
H8A	0.4792	0.5303	0.6466	0.045*	
C9	0.3472 (3)	0.2167 (5)	0.67313 (19)	0.0368 (6)	
C10	0.3059 (3)	0.1160 (6)	0.5631 (2)	0.0438 (6)	
C11	0.1788 (5)	−0.2037 (8)	0.4633 (3)	0.0797 (11)	
H11A	0.1137	−0.3457	0.4753	0.120*	
H11B	0.2749	−0.2545	0.4304	0.120*	
H11C	0.1229	−0.0904	0.4149	0.120*	

### Atomic displacement parameters (Å^2^)

**Table:**

	*U*^11^	*U*^22^	*U*^33^	*U*^12^	*U*^13^	*U*^23^
N1	0.0291 (9)	0.0382 (11)	0.0349 (9)	0.0038 (10)	0.0041 (7)	0.0054 (10)
F1	0.0558 (9)	0.0504 (10)	0.0709 (10)	0.0116 (9)	0.0070 (8)	0.0213 (9)
O1	0.0899 (15)	0.0602 (15)	0.0430 (10)	−0.0071 (15)	0.0099 (10)	−0.0047 (11)
C1	0.0411 (13)	0.0375 (15)	0.0441 (13)	0.0042 (12)	0.0002 (10)	−0.0002 (12)
F2	0.0514 (9)	0.0533 (11)	0.0742 (11)	0.0048 (9)	0.0064 (8)	0.0239 (10)
N2	0.0395 (10)	0.0474 (14)	0.0406 (11)	−0.0038 (11)	0.0050 (9)	0.0102 (11)
O2	0.0674 (12)	0.0578 (14)	0.0587 (11)	−0.0161 (13)	0.0018 (10)	−0.0111 (12)
C2	0.0450 (14)	0.0477 (17)	0.0580 (15)	−0.0094 (14)	0.0106 (12)	−0.0005 (15)
N3	0.0398 (11)	0.0470 (13)	0.0442 (11)	−0.0052 (11)	0.0038 (9)	0.0076 (11)
C3	0.0364 (13)	0.0589 (19)	0.0630 (17)	−0.0032 (15)	0.0059 (12)	−0.0071 (16)
C4	0.0367 (13)	0.0536 (18)	0.0644 (16)	0.0064 (15)	−0.0027 (12)	−0.0005 (17)
C5	0.0380 (12)	0.0394 (15)	0.0451 (12)	0.0027 (13)	0.0018 (10)	0.0046 (13)
C6	0.0335 (11)	0.0396 (13)	0.0353 (11)	0.0012 (12)	0.0013 (9)	−0.0037 (11)
C7	0.0361 (11)	0.0383 (14)	0.0377 (11)	0.0041 (12)	0.0033 (9)	−0.0012 (11)
C8	0.0381 (11)	0.0386 (13)	0.0361 (11)	0.0020 (12)	0.0076 (9)	0.0045 (12)
C9	0.0316 (10)	0.0386 (14)	0.0403 (12)	0.0033 (11)	0.0042 (9)	0.0014 (12)
C10	0.0438 (13)	0.0389 (14)	0.0489 (15)	0.0073 (13)	0.0051 (11)	−0.0037 (13)
C11	0.098 (3)	0.068 (3)	0.073 (2)	−0.016 (2)	−0.0032 (19)	−0.025 (2)

### Geometric parameters (Å, °)

**Table:**

N1—C8	1.339 (3)	C3—C4	1.372 (4)
N1—N2	1.346 (3)	C3—H3B	0.9300
N1—C7	1.461 (3)	C4—C5	1.377 (3)
F1—C1	1.355 (3)	C4—H4A	0.9300
O1—C10	1.198 (3)	C5—C6	1.378 (4)
C1—C2	1.375 (4)	C6—C7	1.507 (3)
C1—C6	1.376 (4)	C7—H7A	0.9700
F2—C5	1.353 (3)	C7—H7B	0.9700
N2—N3	1.300 (3)	C8—C9	1.360 (4)
O2—C10	1.327 (4)	C8—H8A	0.9300
O2—C11	1.439 (4)	C9—C10	1.471 (4)
C2—C3	1.376 (5)	C11—H11A	0.9600
C2—H2B	0.9300	C11—H11B	0.9600
N3—C9	1.362 (3)	C11—H11C	0.9600
			
C8—N1—N2	110.6 (2)	C1—C6—C7	122.9 (2)
C8—N1—C7	129.0 (2)	N1—C7—C6	111.9 (2)
N2—N1—C7	120.43 (18)	N1—C7—H7A	109.2
F1—C1—C2	118.4 (3)	C6—C7—H7A	109.2
F1—C1—C6	117.9 (2)	N1—C7—H7B	109.2
C2—C1—C6	123.7 (3)	C6—C7—H7B	109.2
N3—N2—N1	107.75 (18)	H7A—C7—H7B	107.9
C10—O2—C11	116.1 (3)	N1—C8—C9	104.6 (2)
C1—C2—C3	118.1 (3)	N1—C8—H8A	127.7
C1—C2—H2B	120.9	C9—C8—H8A	127.7
C3—C2—H2B	120.9	C8—C9—N3	108.9 (2)
N2—N3—C9	108.2 (2)	C8—C9—C10	126.8 (2)
C4—C3—C2	121.1 (2)	N3—C9—C10	124.3 (2)
C4—C3—H3B	119.4	O1—C10—O2	124.5 (3)
C2—C3—H3B	119.4	O1—C10—C9	122.8 (3)
C3—C4—C5	118.0 (3)	O2—C10—C9	112.7 (2)
C3—C4—H4A	121.0	O2—C11—H11A	109.5
C5—C4—H4A	121.0	O2—C11—H11B	109.5
F2—C5—C6	117.3 (2)	H11A—C11—H11B	109.5
F2—C5—C4	118.9 (2)	O2—C11—H11C	109.5
C6—C5—C4	123.8 (3)	H11A—C11—H11C	109.5
C5—C6—C1	115.3 (2)	H11B—C11—H11C	109.5
C5—C6—C7	121.8 (2)		
			
C8—N1—N2—N3	0.0 (3)	C8—N1—C7—C6	−59.3 (3)
C7—N1—N2—N3	−179.3 (2)	N2—N1—C7—C6	119.9 (2)
F1—C1—C2—C3	−179.8 (2)	C5—C6—C7—N1	−79.4 (3)
C6—C1—C2—C3	1.1 (4)	C1—C6—C7—N1	100.6 (3)
N1—N2—N3—C9	0.1 (3)	N2—N1—C8—C9	−0.2 (3)
C1—C2—C3—C4	−0.7 (5)	C7—N1—C8—C9	179.1 (2)
C2—C3—C4—C5	0.3 (4)	N1—C8—C9—N3	0.2 (3)
C3—C4—C5—F2	180.0 (2)	N1—C8—C9—C10	−176.5 (2)
C3—C4—C5—C6	−0.3 (4)	N2—N3—C9—C8	−0.2 (3)
F2—C5—C6—C1	−179.7 (2)	N2—N3—C9—C10	176.6 (2)
C4—C5—C6—C1	0.6 (4)	C11—O2—C10—O1	3.4 (4)
F2—C5—C6—C7	0.4 (4)	C11—O2—C10—C9	−176.1 (3)
C4—C5—C6—C7	−179.3 (3)	C8—C9—C10—O1	1.0 (4)
F1—C1—C6—C5	179.9 (2)	N3—C9—C10—O1	−175.3 (3)
C2—C1—C6—C5	−1.0 (4)	C8—C9—C10—O2	−179.4 (2)
F1—C1—C6—C7	−0.2 (4)	N3—C9—C10—O2	4.3 (3)
C2—C1—C6—C7	178.9 (3)		

### Hydrogen-bond geometry (Å, °)

**Table:**

*D*—H···*A*	*D*—H	H···*A*	*D*···*A*	*D*—H···*A*
C7—H7B···N3^i^	0.97	2.62	3.538 (4)	157
C8—H8A···O1^ii^	0.93	2.35	3.243 (3)	162

Symmetry codes: (i) *x*, *y*+1, *z*; (ii) −*x*+1, *y*+1/2, −*z*+1.
